# Comparison of knee flexor strength recovery between semitendinosus alone versus semitendinosus with gracilis autograft for ACL reconstruction: a systematic review and meta-analysis

**DOI:** 10.1186/s12891-024-07226-2

**Published:** 2024-02-12

**Authors:** Angelo Matteucci, Johan Högberg, Ramana Piussi, Mathias Wernbom, Edoardo Franceschetti, Umile Giuseppe Longo, Kristian Samuelsson, Johan Lövgren, Eric Hamrin Senorski

**Affiliations:** 1 Università Cattolica del Sacro Cuore, Largo Francesco Vito 1, 00168 Rome, Italy; 2 Sportrehab Sports Medicine Clinic, Stampgatan 14, 411 01 Gothenburg, Sweden; 3 Sahlgrenska Sports Medicine Center, Sahlgrenska Academy, Gothenburg, Sweden; 4 Unit of Physiotherapy, Department of Health and Rehabilitation, Institute of Neuroscience and Physiology, Sahlgrenska Academy, University of Gothenburg, Box 455, 405 30 Gothenburg, Sweden; 5 Fondazione Policlinico Campus Bio-Medico, Via Alvaro del Portillo 200, Rome, Italy; 6 Research Unit of Orthopaedic and Trauma Surgery, Department of Medicine and Surgery, Università Campus Bio-Medico, Via Alvaro del Portillo 21, 00128 Rome, Italy; 7 Department of Orthopaedics, Institute of Clinical Sciences, Sahlgrenska Academy, University of Gothenburg, Gothenburg, Sweden; 8 Department of Orthopaedics, Sahlgrenska University Hospital, Mölndal, Sweden; 9 Active Physio Sports Medicine Clinic, Brogatan 23, 431 30 Gothenburg, Sweden; 10 Swedish Olympic Committee, Stockholm, Sweden

**Keywords:** Hamstring tendon autograft, Semitendinosus, Gracilis, ACL reconstruction, Knee flexor strength, Assessment

## Abstract

**Background:**

Whether there is a difference in harvesting the semitendinosus tendon alone (S) or in combination with the gracilis tendon (SG) for the recovery of knee flexor strength after anterior cruciate ligament (ACL) reconstruction remains inconclusive. Therefore, this study aimed to assess the recovery of knee flexor strength based on the autograft composition, S or SG autograft at 6, 12, and ≥ 24 months after ACL reconstruction.

**Methods:**

A systematic review and meta-analysis was conducted following the PRISMA guidelines. A comprehensive search was performed encompassing the Cochrane Library, Embase, Medline, PEDRo and AMED databases from inception to January 2023. Inclusion criteria were human clinical trials published in English, comprised of randomized controlled trials (RCTs), longitudinal cohort-, cross-sectional and case–control studies that compared knee flexor strength recovery between S and SG autografts in patients undergoing primary ACL reconstruction. Isokinetic peak torques were summarized for angular velocities of 60°/s, 180°/s, and across all angular velocities, assessed at 6, 12, and ≥ 24 months after ACL reconstruction. A random-effects model was used with standardized mean differences and 95% confidence intervals. Risk of bias was assessed with the RoBANS for non-randomized studies and the Cochrane RoB 2 tool for RCTs. Certainty of evidence was appraised using the GRADE working group methodology.

**Results:**

Among the 1,227 patients from the 15 included studies, 604 patients received treatment with S autograft (49%), and 623 received SG autograft (51%). Patients treated with S autograft displayed lesser strength deficits at 6 months across all angular velocities d = -0.25, (95% CI -0.40; -0.10, *p* = 0.001). Beyond 6 months after ACL reconstruction, no significant difference was observed between autograft compositions.

**Conclusion:**

The harvest of S autograft for ACL reconstruction yields superior knee flexor strength recovery compared to SG autograft 6 months after ACL reconstruction, irrespective of angular velocity at isokinetic testing. However, the clinical significance of the observed difference in knee flexor strength between autograft compositions at 6 months is questionable, given the very low certainty of evidence and small effect size. There was no significant difference in knee flexor strength recovery between autograft compositions beyond 6 months after ACL reconstruction.

**Trial registration:**

CRD42022286773.

**Supplementary Information:**

The online version contains supplementary material available at 10.1186/s12891-024-07226-2.

## Introduction

Anterior cruciate ligament (ACL) reconstruction is performed to restore knee-joint stability in individuals who have sustained an ACL rupture [1, 2]. The hamstring tendon (HT) autograft is, to date, the most widely adopted choice globally for ACL reconstruction [3]. To attain an adequate graft diameter [4], surgeons often use a quadruple-stranded semitendinosus autograft or a combined double-stranded semitendinosus and gracilis tendon autograft [5]. The preference to use a HT autograft rather than a bone-patellar tendon-bone (BPTB) autograft in ACL reconstruction primarily arises from concerns for the risk of persistent anterior knee pain in the short and mid-term, a commonly reported complication after BPTB autograft after ACL reconstruction [6]. Moreover, studies have indicated a greater incidence of knee extensor strength deficits subsequent to ACL reconstruction when BPTB or quadriceps tendon autografts are used for ACL reconstruction compared to HT autografts [7–9]. Conversely, the use of HT autografts has been associated with greater knee flexor strength deficits [7, 8], which potentially negatively influence the knee flexors’ role as a synergistic support to the ACL by mitigating excessive anterior tibial translation, induced by the knee extensors [10]. The harvest of the gracilis tendon in conjunction with the semitendinosus tendon may further affect knee flexor strength compared to harvesting the semitendinosus tendon alone. This is attributed to the gracilis muscle’s contribution to knee flexion strength, particularly at deeper knee angles [11, 12]. In cases where the semitendinosus tendon is used alone for ACL reconstruction, the gracilis muscle has been reported to undergo hypertrophy to compensate for a weakened semitendinosus [13]. Consequently, the harvest of both the gracilis and semitendinosus tendons could potentially lead to a delayed or lack of recovery of knee flexor strength, thereby prolonging the patients’ rehabilitation. According to the current literature, data suggests that patients will have greater knee flexor strength deficits in cases when ACL reconstruction is performed with the semitendinosus tendon in combination with the gracilis tendon compared with a semitendinosus tendon alone [14–16]. However, these findings are limited by few included studies and patients in previous systematic reviews and meta-analysis [14–16].

The objective of this study was to compare knee flexor strength recovery depending on the autograft composition use in ACL reconstruction, specifically by comparing semitendinosus tendon alone (S; regardless of the number of strands and diameter) with the combined semitendinosus tendon and the gracilis tendon (SG) autograft at 6, 12, and ≥ 24 months after ACL reconstruction.

## Methods

This systematic review and meta-analysis adhered to the guidelines outlined in the Preferred Reporting Items for Systematic Reviews and Meta-analyses (PRISMA) [17]. The present systematic review constituted a sub-analysis derived from a broader systematic review prospectively registered in the International Prospective Register of Systematic Reviews (PROSPERO) under registration ID CRD42022286773.

### Eligibility criteria

Original studies with the following characteristics were considered eligible for inclusion:

1. Studies written in English.
2. Cross-sectional studies, prospective and retrospective cohort studies, case–control studies, and randomized clinical trials (RCTs) without limitations on publication timeframe.
3. Studies on patients who underwent primary ACL reconstruction and compared S with SG autografts harvested from the ipsilateral side.
4. Studies that evaluated isokinetic knee flexor strength and presented the strength either as a deficit in comparison to the uninjured side or as limb symmetry index (LSI).

The following characteristics led to the exclusion of studies:

1. Case reports, reviews, letters to the editor, commentaries, and editorials.
2. Studies on a paediatric population (where all patients were < 16 years old).
3. Studies on patients with a previous ACL injury on the contralateral side.
4. Studies which could not be attained in full text.

### Information sources and search strategy

A systematic search was performed by a medical librarian from the Biomedical Library at the University of Gothenburg in December 2021. An update to this search was conducted in January 2023 using the following databases: Cochrane, Excerpta Medica database (EMBASE), Medical Literature Analysis and Retrieval System Online (Medline), Allied and Complementary Medicine Database (AMED) and Physiotherapy Evidence Database (PEDro). The search string combined the use of Medical Subject Headings (MeSH) and free text terms including Anterior Cruciate Ligament, ACL, surgical, surgery, surgeries, reconstruction, reconstructive, reconstructed, repair, anterior cruciate ligament reconstruction, hamstring tendons, hamstring muscles, semitendinosus, graft, autograft, treatment outcome, recovery of function, range of motion, articular, muscle, flexor, strength, hamstring, transplant, tissue, and flexion. A similar search strategy was used with adaptation to each database configuration (Supplemental files 1 and 2).

### Selection process

Two authors (JH and JL) independently reviewed all titles and abstracts to assess eligibility using the Rayyan QCRI web application for systematic reviews [18]. Studies deemed eligible after initial screening of titles and abstracts were subsequently reviewed in full text to confirm eligibility with the inclusion criteria before being considered for inclusion. The Cohen’s kappa coefficient displayed an agreement of 88%. Additionally, reference lists of identified reviews from the systematic search were screened to identify eventual additional studies. Any uncertainties or disagreements between the two authors was solved through a discussion with the senior author (EHS).

### Data collection process

Data from the included studies was extracted into an Excel spreadsheet (version 16; Microsoft Corporation, Redmond, Washington, USA) by the first and second author (AM and JH). In case of uncertainty or disagreement, a consensus discussion was held together with the senior author (EHS) to ensure accuracy and consistency.

### Data items

Extracted data consisted of the following:

1. Study information: author, publication year, title, journal, study type, purpose, and main conclusions.
2. Population details: sample size, sex distribution, age, sport involvement, sport level, and the type of autograft used (S or SG autograft).
3. Methodology specifics: test apparatus (e.g., Biodex or Cybex), assessment mode (isokinetic), contraction type (concentric/eccentric), range of motion, angular velocity, number of repetitions, and rest between sets.
4. Information regarding assessment of knee flexor strength: timepoint of assessment after ACL reconstruction, relative strength deficit and LSI.

The primary outcome of interest was knee flexor strength, specifically presented as the relative strength deficit among patients undergoing ACL reconstruction with either S or SG autografts at 6, 12, and ≥ 24 months after ACL reconstruction. The relative strength deficit was presented as a proportional difference compared to the uninjured limb. Positive values denoted a deficit in knee flexor strength in relation to the uninjured limb, whereas negative values indicated that the injured side exhibited greater strength than the uninjured limb. For instance, if the injured leg displayed 130 Newton meters (Nm) and the uninjured 150 Nm in knee flexor strength, the deficit would be presented as 13% (130/150 = 0.87). While some studies directly reported the relative strength, others used the LSI. The LSI represents the relative strength, calculated by dividing the injured limb’s result by the uninjured limb’s result, then multiplying by 100 to express it as a percentage, with the uninjured limb considered as the reference of “100%” [19]. To strive for homogeneity, the LSI was recalculated to relative strength deficits by subtracting the reported LSI value from 100%, (e.g., 92% for LSI became 8%) and maintained the same standard deviation as reported for the LSI. This allowed for uniformity in the representation of relative strength deficits across studies.

### Risk of bias assessment

Two authors (JH and RP) performed a risk of bias assessment for non-randomized studies using the Risk of Bias Assessment Tool for Non-Randomized Studies (RoBANS) [20]. The RoBANS comprises six domains: a) patient selection, b) confounding variables, c) measurement of exposure, d) blinding of the outcome assessments, e) incomplete outcome data, and f) selective outcome reporting. Each domain was categorized as low risk, high risk, or unclear risk of bias [20].

For the assessment of the RCTs, the Cochrane Risk of Bias (RoB) 2 tool was used [21]. The Cochrane RoB 2 includes five domains: 1) risk of bias related to the randomization process, 2) bias arising from deviations in the intended intervention, 3) missing outcome data, 4) bias in outcome measurement, and 5) bias in selection of the reported result. Each domain contains signal questions designed to gather information pertinent to bias assessment, with possible answers such as “yes”, “probably yes”, “probably no”, “no”, and “no information”.

The following interpretation of risk of bias was used:

- Low risk of bias: All domains were judged as having low risk of bias.
- Some concerns: No domain was judged as high risk but at least one domain raised some concerns.
- High risk of bias: At least one domain was rated as high risk, or the study exhibited some concerns in multiple domains that substantially lowered the confidence in the result [21].

### Effect measures

Effect sizes were computed as standardized mean differences to facilitate the aggregation of data for the comparison between S and SG autografts. The standardized mean difference was calculated by the difference between mean scores between S and SG autografts, divided by the pooled standard deviation. Interpretation of the standardized mean difference used the following reference benchmarks: 0.2 to 0.5 = a small effect, 0.5 to 0.8 = a moderate effect, and > 0.8 = a large effect [22].

### Data synthesis

Patients from the included studies were categorized into two groups based on the type of autograft used: S autograft or SG autograft. The relative knee flexor strength deficits were presented as mean values along with standard deviations and were converted into standardized mean differences along with 95% confidence intervals. The standardized mean differences were then pooled and visualized using forest plots generated in Review Manager software (RevMan, version 5.4.1, Cochrane, London, UK) for different angular velocities (60°/s, 180°/s, and all velocities) at 6, 12, and ≥ 24 months after ACL reconstruction. Studies within ± 1 months of the specified follow-up durations (6 and 12 months) were included. Data concerning knee flexor strength assessments that could not be pooled, e.g., at other timepoints, in other positions than seated, eccentric assessment, or data presented for specified knee angles were qualitatively summarized in Table 4. The degree of heterogeneity in knee flexor strength between included studies was assessed with the I^2^ index, which was interpretated as follows: 0.0–24.9% to indicate no heterogeneity, 25.0–49.9% to indicate low heterogeneity; 50.0–74.9% to indicate moderate heterogeneity; 75.0–100.0% to indicate high heterogeneity [23]. A higher I^2^ score implies a larger proportion of variability in the results could be attributed to heterogeneity [23]. Clinical heterogeneity was assessed through author discussions, noting moderate to high clinical variation in knee flexor strength assessment methodologies. Consequently, random effects models were applied, even in cases where I^2^ indicated low or no heterogeneity. To address differences in sample size across studies, weighted mean values were used instead of arithmetic mean values. The use of weighted mean values allowed studies with larger sample sizes to contribute more to the computed average compared to studies with small samples sizes.

### Certainty assessment

The certainty of evidence for the outcome of interest was evaluated by two authors (JH and RP) using the Grading of Recommendations Assessment Development and Evaluation (GRADE) working group methodology [24]. Study quality was categorized as “high” for RCTs, and “low” for observational studies. When both types of studies were included for the outcome of interest, the overall study quality was considered “low”. After the study quality was determined based on the included studies’ design, potential downgrades in the certainty of evidence (by one or two levels, such as from high to moderate or high to low) were considered based on:

1. Study limitations: Serious risk of bias assessed via RoB 2 or RoBANS [20, 21].
2. Inconsistency: Substantial heterogeneity evaluated by the I^2^-index.
3. Indirectness: Poor generalizability due to differences in population, knee flexor strength assessment methodologies, and/or different outcome measures.
4. Imprecision: Wide confidence intervals upon pooling or small sample sizes.
5. Risk of publication bias: Evaluated through funnel plots.
6. In instances of a substantial standardized mean difference, larger sample sizes, and limited dispersion, the certainty of evidence could potentially be upgraded by one level.

Taking into account study quality, limitations, inconsistency, indirectness, imprecision, risk of publication bias, substantial mean differences, larger sample sizes, and limited dispersion, the certainty of evidence could be graded as high, moderate, low, or very low.

## Results

### Study selection

**Fig. 1:**
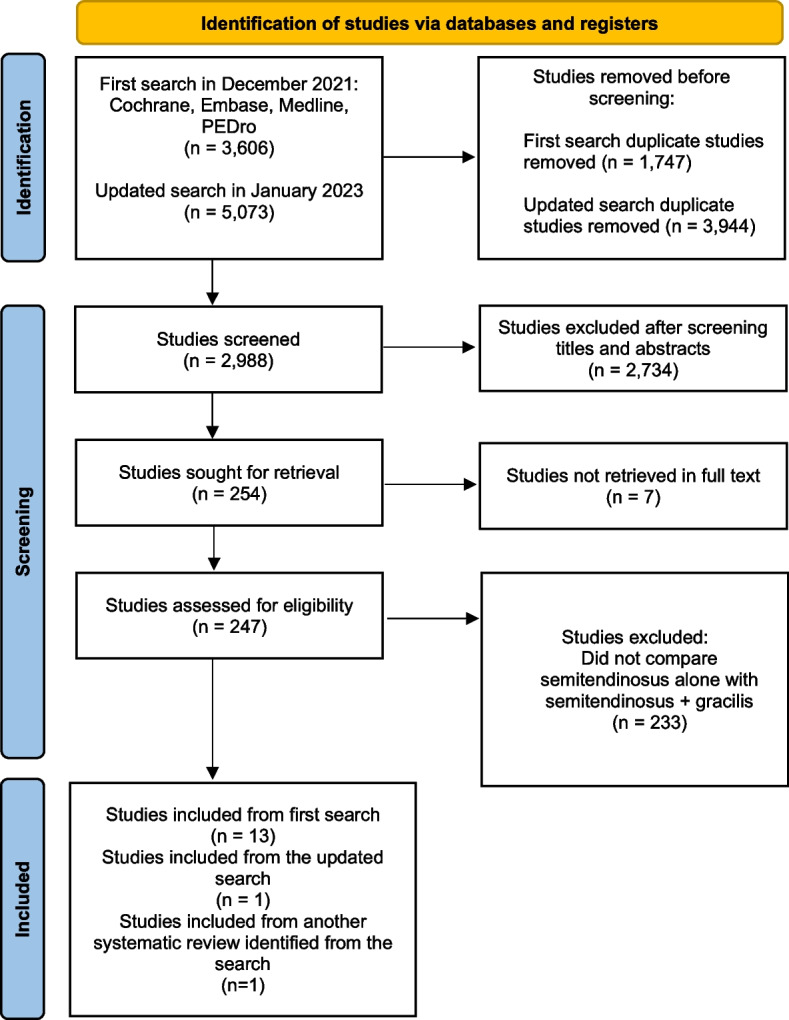
PRISMA (Preferred Reporting Items for Systematic Reviews and Meta-Analyses) flow diagram of included and excluded studies

The initial search yielded 3,606 studies, with 1,747 identified as duplicates. Following the updated search, 5,073 studies were found, of which 3944 were duplicates. Subsequently, 2,988 studies were screened based on their titles and abstracts, leading to 247 studies being read in full text. Finally, 15 studies met the inclusion criteria. Figure 1 illustrates the selection process.

### Study characteristics

Data from 1,227 patients originating from 15 studies, published between years 1999 and 2022, were extracted. Among the included patients, 604 underwent treatment with S (49%) autograft, and 623 received SG (51%) autograft. The included individuals consisted of 892 male patients (73%), 267 female patients (22%), and 68 cases (5%) where sex information was not reported. Across the included studies, the average age ranged from 19.6 ± 7 to 32.5 ± 6.7 years old. Table 1 provides an overview of the characteristics of the included studies.

**Table 1:** Overview of included studies

Author	Study design/ Level of evidence	Autograft (n, %)	Sex (M/W) (W, %)	Age (Mean ± SD)	Assessment time (Months)
Adachi (2003) [25]	Prospective cohort study Level II	**S:** 26 (59%) **SG:** 18 (41%) **Total:** 44	**S:** 15/11 **SG:** 12/6 **Total:** 17 (39%)	**S:** 27.7 ± 10.5 **SG:** 25.6 ± 8.9	**Both:** 12, 35
Ardern (2010) [26]	Retrospective cohort study Level III	**S:** 20 (40%) **SG:** 30 (60%) **Total:** 50	**S:** 15/5 **SG:** 20/10 **Total:** 15 (30%)	**S:** 27.2 ± 5 **SG:** 28.7 ± 7	**S:** 33.5 ± 4.1 **SG:** 31.4 ± 7
Barenius (2013) [27]	Retrospective cohort study Level III	**S:** 10 (50%) **SG:** 10 (50%) **Total:** 20	**S:** 8/2 **SG:** 8/2 **Total:** 4 (25%)	**S:** 26 ± 7 **SG:** 26 ± 9	**S:** 36 ± 4 **SG:** 37 ± 6
Carter (1999) [28]	Randomized controlled trial Level I	**S:** 33 (49%) **SG:** 35 (51%) **Total:** 68			**Both:** 6
de Geofroy (2022) [29]	Retrospective cohort study Level III	**S:** 69 (52%) **SG:** 64 (48%) **Total:** 133	**S:** 66/3 **SG:** 63/1 **Total:** 4 (3%)	**S:** 28.5 ± 6.2 **SG:** 29.8 ± 6.5	**Both:** 4, 8
Drocco (2017) [30]	Retrospective cohort study Level III	**S:** 45 (50%) **SG:** 45 (50%) **Total:** 90	**S:** 34/11 **SG:** 27/18 **Total**: 29 (32%)	**S:** 29.5 ± 10 **SG:** 27.7 ± 9	**Both:** 6
Gillet (2022) [31]	Retrospective cohort study Level III	**S:** 67 (36%) **SG:** 119 (64%) **Total:** 186	**S:** 67/0 **SG:** 119/0 **Total:** 0 (0%)	**S:** 26.9 ± 6.4 **SG:** 25.6 ± 6.1	**Both:** 6
Inagaki (2013) [32]	Prospective cohort study Level II	**S:** 61 (51%) **SG:** 59 (49%) **Total:** 120	**S:** 35/26 **SG:** 33/26 **Total:** 52 (43%)	**S:** 28.2 ± 11.9 **SG:** 26.2 ± 10.3	**Both:** 24
Kouloumentas (2019) [33]	Randomized controlled trial Level I	**S:** 45 (50%) **SG:** 45 (50%) **Total:** 90	**S:** 28/17 **SG:** 27/18 **Total:** 35 (39%)	**S:** 27.6 ± 11.4 **SG:** 29.7 ± 11.0	**Both:** 24
Lee (2019) [34]	Retrospective cohort study Level III	**S:** 60 (50%) **SG:** 60 (50%) **Total:** 120	**S:** 57/3 **SG:** 55/5 **Total:** 8 (7%)	**S:** 27.4 ± 6.6 **SG:** 26.9 ± 7.3	**S:** 37.5 ± 5.9 **SG:** 36.8 ± 6.1
Monaco (2018) [35]	Retrospective cohort study Level III	**S:** 22 (50%) **SG:** 22 (50%) **Total:** 44	**S:** 15/7 **SG:** 17/5 **Total:** 12 (27%)	**S:** 32.5 ± 6.7 **SG:** 31.7 ± 7.1	**Both:** 13 (12–14)
Nakamura (2002) [36]	Case–control study Level III	**S:** 49 (66%) **SG:** 25 (34%) **Total:** 74	**S:** 28/21 **SG:** 6/19 **Total:** 40 (54%)	**S:** 24.3 **SG:** 25.7	**Both:** 24
Roger (2020) [37]	Randomized controlled trial Level I	**S:** 33 (55%) **SG:** 27 (45%) **Total:** 60	**S:** 26/7 **SG:** 23/4 **Total:** 11 (18%)	**S:** 30.5 ± 8.9 **SG:** 30.3 ± 8.5	**Both:** 6, 24
Sengoku (2022) [38]	Retrospective cohort study Level III	**S:** 41 (50%) **SG:** 41 (50%) **Total:** 82	**S:** 21/20 **SG:** 21/20 **Total:** 40 (49%)	**S:** 21.7 ± 9.2 **SG:** 19.6 ± 7	**Both:** 3, 6
Yosmaoglu (2011) [39]	Prospective cohort study Level II	**S:** 23 (50%) **SG:** 23 (50%) **Total:** 46	**S:** 23/0 **SG:** 23/0 **Total:** 0 (0%)	**S:** 29 ± 7 **SG:** 28 ± 9	**Both:** 12

*M* men, *n* numbers of individuals, *SD* Standard deviation, *S* Semitendinosus, *SG* Semitendinosus + gracilis, *W* Women

### Risk of bias assessment of non-randomized controlled trials

In the selection of studies, 12 out of the 15 included (80%) were non-randomized controlled trials. The most prevalent issue associated with high risk of bias was the absence of accounting for confounding variables. Table 2 outlines the RoBANS assessment.

**Table 2:**
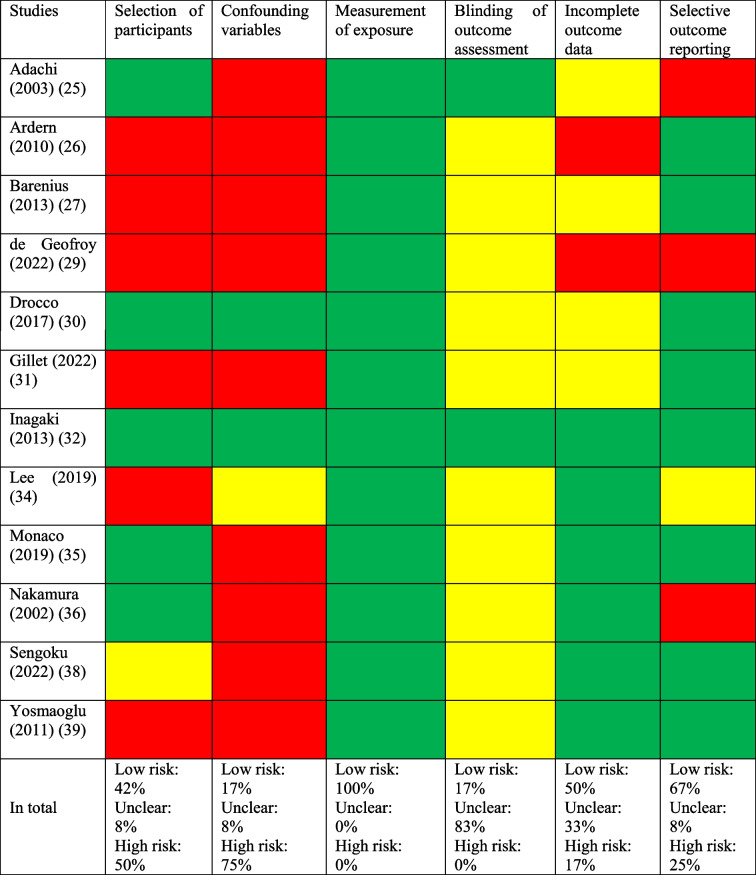
Risk of bias assessed with risk of bias assessment tool for non-randomized studies

Green=Low risk of bias; Yellow=Unclear; Red=High risk of bias

### Risk of bias assessment of randomized controlled trials

Out of the 15 studies included, three (20%) were RCTs. Among these trials, one was categorized as having a high risk of bias, one raised some concerns regarding bias, and one was deemed to have low risk of bias. The primary methodological weakness commonly identified was associated with the selection of reported results. Table 3 illustrates the risk of bias assessment using the Cochrane RoB 2 tool.

**Table 3:**
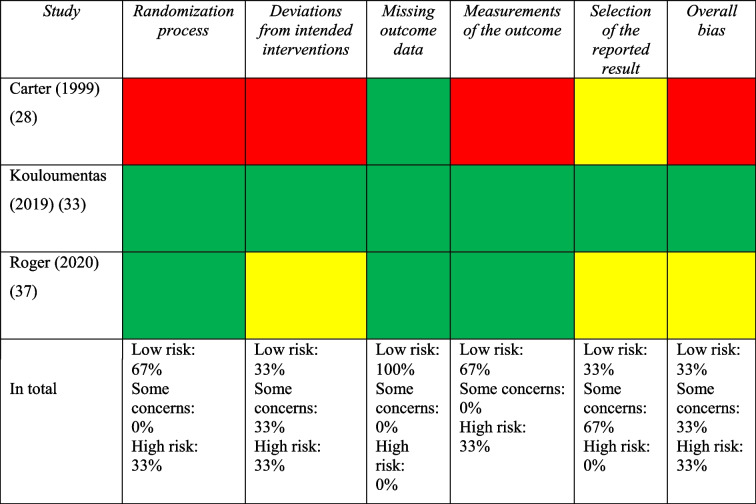
Risk of bias assessed with cochrane risk of bias tool for randomized controlled trials

Green=Low risk of bias; Yellow=Some concerns; Red=High risk of bias

### Result of the quantitative synthesis

#### The 6-month follow-up

**Fig. 2:**
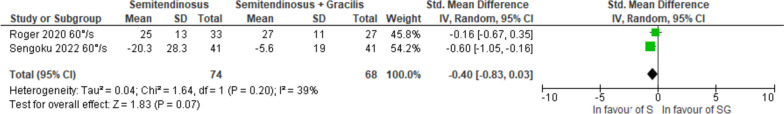
Pooled results for the knee flexor strength assessed isokinetic with an angular velocity of 60°/s between semitendinosus alone (S) and semitendinosus with gracilis (SG) autografts at 6 months follow-up

**Fig. 3:**
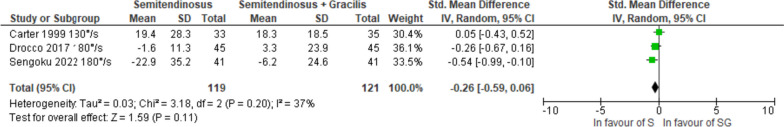
Pooled results for the knee flexor strength assessed isokinetic with an angular velocity of 180°/s between semitendinosus alone (S) and semitendinosus with gracilis (SG) autografts at 6 months follow-up

**Fig. 4:**
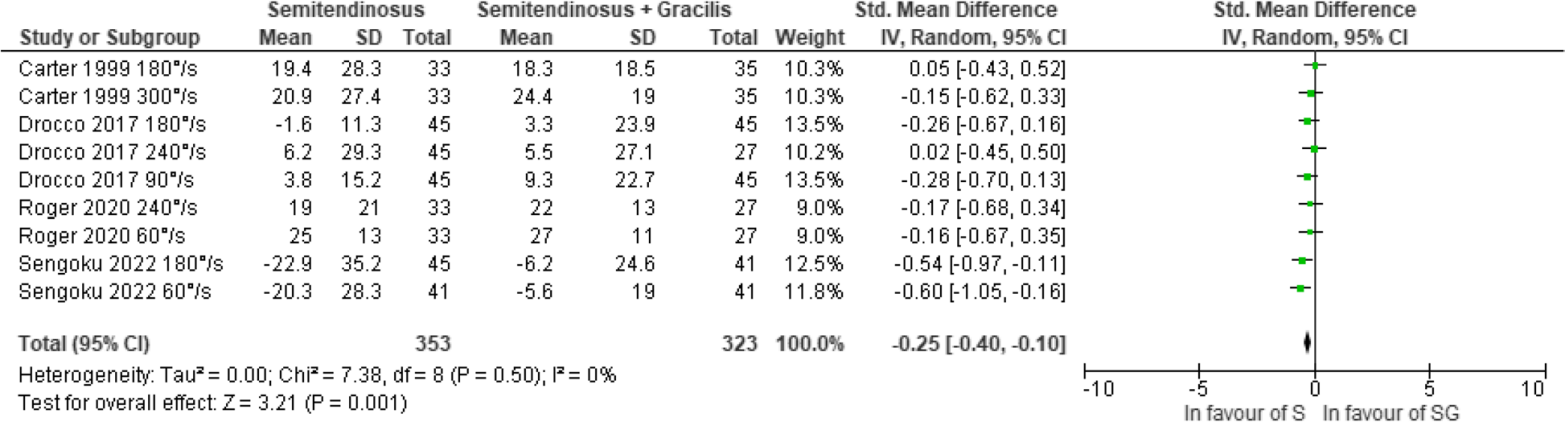
Pooled results for the knee flexor strength assessed isokinetic regardless of angular velocity between semitendinosus alone (S) and semitendinosus with gracilis (SG) autografts at 6 months follow-up

With a very low certainty of evidence, no significant difference in knee flexor strength recovery was observed between S and SG autografts when examined separately at angular velocities of 60°/s [37, 38] and 180°/s [28, 30, 38] (Figs. 2 and 3). With a very low certainty of evidence, patients treated with S autograft displayed lesser strength deficits at 6 months when considering all angular velocities d = -0.25, (95% CI -0.40; -0.10, *p* = 0.001) (Fig. 4) [28, 30, 37, 38]. The certainty of evidence was downgraded due to risk of bias and publication bias. There was no statistical heterogeneity considering all angular velocities merged (I^2^ = 0%), although a low statistical heterogeneity was observed at both 60°/s (I^2^ = 39%) and at 180°/s (I^2^ = 37%).

#### The 12-month follow-up

**Fig. 5:**
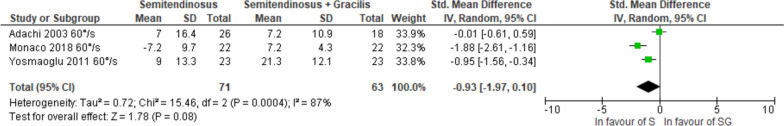
Pooled results for the knee flexor strength assessed isokinetic with an angular velocity of 60°/s between semitendinosus alone (S) and semitendinosus with gracilis (SG) autografts at 12 months follow-up

**Fig. 6:**
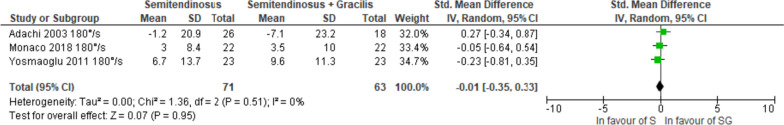
Pooled results for the knee flexor strength assessed isokinetic with an angular velocity of 180°/s between semitendinosus alone (S) and semitendinosus with gracilis (SG) autografts at 12 months follow-up

**Fig. 7:**
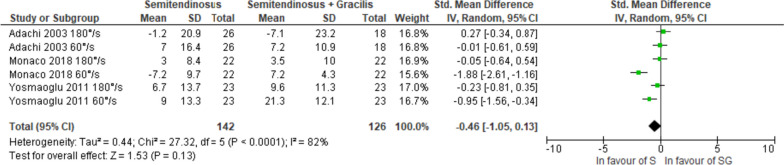
Pooled results for the knee flexor strength assessed isokinetic regardless of angular velocity between semitendinosus alone (S) and semitendinosus with gracilis (SG) autografts at 12 months follow-up

With a very low certainty of evidence, no significant difference in knee flexor strength was observed between S and SG autografts when examined at angular velocities of 60°/s [25, 35, 39], 180°/s [25, 39], or when collectively analysed regardless of angular velocity (Figs. 5, 6 and 7) [25, 35, 39]. The certainty of evidence was downgraded due to risk of bias, inconsistency, and publication bias. There was no statistical heterogeneity considering an angular velocity of 180°/s (I^2^ = 0%), although a high statistical heterogeneity was observed at 60°/s (I^2^ = 87%) and for all regardless angular velocity (I^2^ = 82%).

#### The ≥ 24 months follow-up

**Fig. 8:**
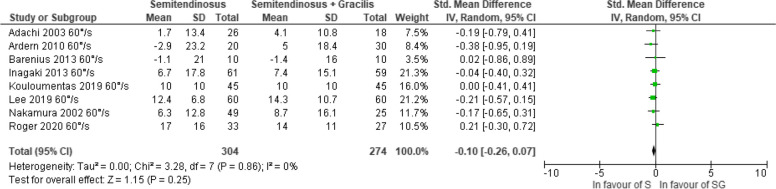
Pooled results for the knee flexor strength assessed isokinetic with an angular velocity of 60°/s between semitendinosus alone (S) and semitendinosus with gracilis (SG) autografts at ≥ 24 months follow-up

**Fig. 9:**
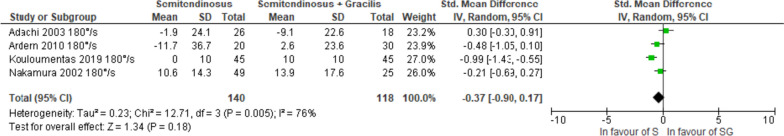
Pooled results for the knee flexor strength assessed isokinetic with an angular velocity of 180°/s between semitendinosus alone (S) and semitendinosus with gracilis (SG) autografts at ≥ 24 months follow-up

**Fig. 10:**
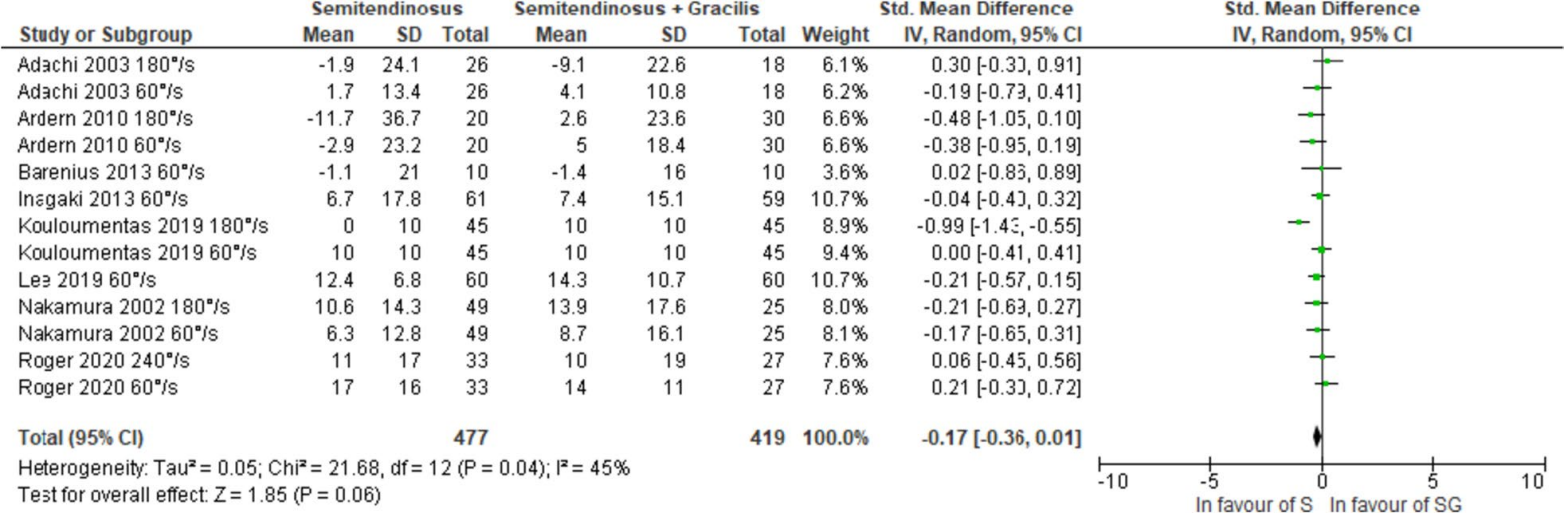
Pooled results for the knee flexor strength assessed isokinetic regardless of angular velocity between semitendinosus alone (S) and semitendinosus with gracilis (SG) autografts at ≥ 24 months follow-up

With a very low certainty of evidence, no significant difference in knee flexor strength recovery was observed between S and SG autografts when assessed at angular velocities of 60°/s [25–27, 32–34, 36, 37], 180°/s [25, 26, 33, 36], or when collectively analysed irrespective of angular velocity (Figs. 8, 9 and 10) [25–27, 32–34, 36, 37]. The certainty of evidence was downgraded due to risk of bias and publication bias. There was no statistical heterogeneity considering an angular velocity of 60°/s (I^2^ = 0%), although a high statistical heterogeneity was observed at an angular velocity of 180°/s (I^2^ = 76%) and a moderate statistical heterogeneity when considering all angular velocities (I^2^ = 45%).

### Result of the qualitative synthesis

Table 4 displays the included studies that assessed isokinetic knee flexor strength conducted at time points other than 6, 12, and ≥ 24 months, evaluations in positions other than seated, eccentric assessment, or reported isokinetic knee flexor strength deficits at specified knee angles rather than the knee flexor strength peak torque. Thus, studies and subgroups summarized in Table 4 involve comparisons between S and SG autografts but are not incorporated into the forest plots.

**Table 4:** Qualitative synthesis of studies comparing semitendinosus alone and semitendinosus with gracilis autografts which were not included in the forest plots due to reporting specified knee angles, other positions than seated, eccentric assessment or at other timepoints than 6, 12 and ≥ 24 months

Author/sub-group	Time for assessment	Position for assessment	Peak torque	30°	60°	90°	105°
Ardern (2010) [26] 60°/s	**S:** 33.5 ± 4.1 **SG:** 31.4 ± 7	Seated			**S: -**30.4 ± 87.0 **SG: -**12.2 ± 99.3	**S:** 14.6 ± 31.6 **SG:** 10.4 ± 29.4	**S:** 21.7 ± 44.7 **SG:** 24.7 ± 40.1
Ardern (2010) [26] 180°/s	**S:** 33.5 ± 4.1 **SG:** 31.4 ± 7	Seated			**S:** -28.6 ± 55.6 **SG: -**8.8 ± 74.4	**S:** -10.3 ± 106.9 **SG:** 21.2 ± 196.1	**S:** -48.0 ± 109.4 **SG:** 13.3 ± 51.9
Barenius (2013) [27] 60°/s	**S:** 36 ± 4 **SG:** 37 ± 6	Seated				**S:** 14.2 ± 30 **SG:** 22.0 ± 36	
de Geofroy (2022) [29] Angular velocity unspecified	**Both:** 4	Unspecified	**S:** 17 **SG:** 26				
de Geofroy (2022) [29] Angular velocity unspecified	**Both:** 8	Unspecified	**S:** 8 **SG:** 22				
Gillet (2022) [31] 90°/s (Range)	**Both:** 6	Seated	**S:** 18.6–21.3 **SG:** 12.3–25.1				
Gillet (2022) [31] 180°/s (Range)	**Both:** 6	Seated	**S:** 10.2–16.2 **SG:** 10.5–21.1				
Gillet (2022) [31] 240°/s (Range)	**Both:** 6	Seated	**S:** 13.9–16.8 **SG:** 11.9–19.0				
Gillet (2022) [31] 30°/s, eccentric (Range)	**Both:** 6	Seated	**S:** 12.5–26.2 **SG:** 5.9–24.2				
Lee (2019) [34] 60°/s	**S:** 37.5 ± 5.9 **SG:** 36.8 ± 6.1	Prone (60°-120°)	**S:** 13.4 ± 8.9 **SG:** 24.2 ± 13.4				
Monaco (2018) [35] 60°/s	**Both:** 13 (12–14)	Seated		**S:** 3.1 ± 10.3 **SG:** -11 ± 11.2			
Monaco (2018) [35] 180°/s	**Both:** 13 (12–14)	Seated		**S:** -7.5 ± 11.2 **SG:** -7.6 ± 15.4			
Nakamura (2002) [36] 60°/s	**Both:** 24	Unspecified				**S:** 19.8 ± 27.5 **SG:** 21.2 ± 21.7	
Nakamura (2002) [36] 180°/s	**Both:** 24	Unspecified				**S:** 10.6 ± 14.3 **SG:** 28.5 ± 30.3	
Sengoku (2022) [38] 60°/s	**Both:** 3	Unspecified	**S:** 8.3 ± 18.7 **SG:** 16.2 ± 21.8				
Sengoku (2022) [38] 180°/s	**Both:** 3	Unspecified	**S:** 4.7 ± 26.9 **SG:** 12.6 ± 21.6				

Positive values indicate a deficit, while negative values indicate that the injured side is stronger than the uninjured side. *s* Seconds, *S* Semitendinosus alone, *SG* Semitendinosus + gracilis

## Discussion

There is a very low certainty of evidence that suggests that the use of a S autograft may lead to better recovery of isokinetic knee flexor strength compared to SG autograft, irrespective of angular velocity at six months after ACL reconstruction. Nevertheless, the effect size was small. Beyond the six months follow-up after ACL reconstruction, there was no significant difference in knee flexor strength recovery between the two graft compositions. Despite the minor deficit in knee flexor strength observed with the use of S autografts compared to SG autografts at the six months follow-up, our results suggest that graft composition does not appear to significantly affect the clinical recovery of knee flexor strength.

The regeneration of the semitendinosus and gracilis tendons after harvest for ACL reconstruction has been documented [40, 41]. Papandrea et al. [42] noted signs of semitendinosus tendon regeneration as early as one month after ACL reconstruction, with ongoing adaptation observed up to 24 months after surgery [42]. As the semitendinosus tendon, and potentially the gracilis tendon, undergoes adaptive changes to regenerate, especially during the first year after ACL reconstruction [42], other knee flexor muscles might have to compensate for the lack of force production when the semitendinosus and gracilis muscles are still too weak. In support of this notion, Tampere et al. [43] suggested that the biceps femoris partly compensated for deficits in semitendinosus function during an eccentric hamstring loading task in patients after ACL reconstruction with S autografts. The reduced strength deficits observed in patients treated with the S autograft compared to patients with the SG autograft (d = -0.25, [95% CI -0.40; -0.10, *p* = 0.001]) in our study at the six months follow-up might suggest the potential role for the gracilis tendon to compensate when the semitendinosus is still not sufficiently robust to generate adequate force. However, the effect size of the difference between S and SG autografts in the present study was small, which raises questions about its clinical relevance. It is plausible that other knee flexor muscles with larger muscle volume, such as the semimembranosus and biceps femoris, contribute more than the gracilis muscle [43].

Consistent with our results, a previous systematic review by Sharma et al. [15] concluded that the use of a SG autograft for ACL autograft led to a 3.9% reduction isokinetic peak knee flexor strength at an angular velocity of 60°/s compared to the use of S autograft based on a minimum two-year follow-up. No significant differences were reported at 180°/s and 240°/s [15], which suggests that the choice of autograft composition might not yield clinically differences in knee flexor strength recovery. A more recent systematic review to date by Chin et al. [16] reported that incorporating the harvest of the SG autograft in ACL reconstruction resulted in greater deficits in knee flexor strength at both 60°/s and 180°/s at the two-year follow-up compared to the S autograft. Overall, the collected data suggests significant deficits in knee flexor strength when ACL reconstruction is performed with gracilis tendon in addition to the semitendinosus tendon, the clinical relevance is however questionable.

In the current meta-analysis, the peak torque of knee flexors was assessed without consideration to specific knee flexion angles. The decision to aggregate knee flexor peak torque measurements might be questioned, as prior studies have suggested that harvesting the gracilis tendon in addition to the semitendinosus tendon could particularly impact knee flexor strength at deeper knee angles [44]. On the other hand, some data indicate that a larger deficit in knee flexor strength at deeper knee angles might not specifically occur in patients with additional gracilis tendon harvest [45]. Similar patterns of greater knee flexor strength deficits at deeper knee angles have been observed in patients who had only the S autograft [45]. Previous studies that have compared knee flexor strength between the S and SG autografts showed no difference in knee flexor strength at deeper knee angles [26, 27]. The knee flexor strength deficit in deeper knee angles appears to be associated to subsequent alternations in hamstring muscle morphology, such as semitendinosus muscle retraction and atrophy following ACL reconstruction with HT autografts [46, 47]. Although a knee flexor strength deficit might persist at deeper knee angles regardless of graft composition, peak torque at lower degrees of knee flexion appears to have a stronger correlation with functional performance compared to deeper knee angles [48, 49]. Hence, one could argue that to consider the overall peak torque of the knee flexors after ACL reconstruction holds more relevance than to focus solely on the peak torque at deeper knee angles. The findings from this meta-analysis suggest a minor influence of HT autograft composition on isokinetic peak torque, showing a smaller knee flexor strength deficit for S autograft compared to SG autograft at six months after ACL reconstruction. However, this difference between graft compositions was not significant beyond the six months follow-up following ACL reconstruction. While the mean deficit was not individually analysed for each autograft in this study, it seems reasonable to not make a difference in rehabilitation for patients after ACL reconstruction depending on graft composition, that is, S or SG autografts.

The decision to combine angular velocities in the meta-analysis deserves consideration. Lower angular velocities (e.g., 60°/s) have been proposed to yield more reliable peak torque values [50]. The advantage of merging results from different angular velocities lies in the potential inclusion of more studies to obtain a larger sample size. As peak torque values may differ across various angular velocities, we conducted separate analyses for 60°/s and 180°/s. The harvest of the SG autograft has been suggested to lead to deficits in knee flexor strength at deeper knee angles [44]. Regrettably, we lacked sufficient data to aggregate knee flexor strength specifically for deeper knee angles between the different autografts. Furthermore, we did not conduct subgroup analyses based on sex. Only 22% of the patient population comprised women in the present systematic review and meta-analysis, thus, limiting the generalizability of our findings for women. Four studies enrolled > 100 patients [29, 31, 32, 34], while the remaining studies included fewer than 100 patients, with the smallest sample size being 20 patients [27]. The varying sample size raise concerns about the risk of bias arising from smaller samples. Confounding factors, such as concomitant meniscal injuries [51] were not addressed in the statistical analysis which could have affected the investigation of the recovery of knee flexor strength. Lastly, there existed a notable risk of bias in both the non-randomized studies and the randomized controlled trials, contributing to a very low certainty of evidence based on the GRADE assessment. This underscores the need for caution in the interpretation of our results.

There is a need for a future large multicenter RCT considering the limitations inherent in both the current and prior systematic reviews and meta-analysis, leading to a lack of generalizability. Such a study is imperative to conclusively determine whether there is a difference in harvesting the semitendinosus alone or in combination with the gracilis tendon for knee flexor strength recovery following ACL reconstruction.

## Conclusion

A very low certainty of evidence indicates that the use of S autograft yields superior results compared to the SG autograft in terms of knee flexor strength recovery, irrespective of angular velocity at isokinetic testing, at six months after ACL reconstruction. However, after six months, there were no difference observed between graft compositions. It is uncertain whether the difference in knee flexor strength between autograft compositions at six months holds clinical significance, given that the effect size was small.

### Supplementary Information

**Additional file 1. Supplemental file 1**.

**Additional file 2. Supplemental file 2**.

## Data Availability

All included studies constituting the present systematic review and meta-analysis are cited in the manuscript. The dataset is available from the corresponding author on a reasonable request.
