# Supplementary material for: Comparison of knee flexor strength recovery between semitendinosus alone versus semitendinosus with gracilis autograft for ACL reconstruction: a systematic review and meta-analysis
Source: BMC Musculoskelet Disord. 2024 Feb 12;25:136. doi: 10.1186/s12891-024-07226-2 (PMC10863077; doi:10.1186/s12891-024-07226-2)
Supplement: Supplementary file 1 — Additional file 1. Supplemental file 1. [file 12891_2024_7226_MOESM1_ESM.docx]

**Supplemental file 1**

First search in December 2021.

**Database:** MEDLINE (OVID)

**Date:** 2021-12-02

**No of results:** 1400 ref

| **#** | **Searches** | **Results** |
| --- | --- | --- |
| 1 | exp Anterior Cruciate Ligament/ | 11,524 |
| 2 | ACL.ab,kw,ti. | 18,291 |
| 3 | (anterior adj5 cruciate adj5 ligament*).ab,kw,ti. | 21,030 |
| 4 | 1 or 2 or 3 | 27,954 |
| 5 | (surgical* or surgery or surgeries or reconstruction* or reconstructive or reconstructed or  repair).ab,kw,ti. | 2,424,589 |
| 6 | 4 and 5 | 17,317 |
| 7 | exp Anterior Cruciate Ligament Reconstruction/ | 6,216 |
| 8 | 6 or 7 | 17,725 |
| 9 | Hamstring Tendons/tr | 374 |
| 10 | Hamstring Muscles/tr | 65 |
| 11 | 9 or 10 | 434 |
| 12 | (Hamstring or semitendinosus).ab,kw,ti. | 10,251 |
| 13 | (graft or autograft or transplant* or tissue).ab,kw,ti. | 2,074,827 |
| 14 | 12 and 13 | 3,608 |
| 15 | 11 or 14 | 3,694 |
| 16 | exp treatment outcome/ | 1,160,264 |
| 17 | exp "Recovery of Function"/ | 57,273 |
| 18 | exp "Range of Motion, Articular"/ | 56,683 |
| 19 | ("range of motion" or recover* or outcome or function* or flexion or extension).ab,kw,ti. | 5,618,085 |
| 20 | 16 or 17 or 18 or 19 | 6,384,571 |
| 21 | (Muscle or flexor).ab,kw,ti. | 693,757 |
| 22 | strength.ab,kw,ti. | 325,889 |
| 23 | 21 and 22 | 47,448 |
| 24 | 20 or 23 | 6,402,851 |
| 25 | 8 and 15 and 24 | 1,573 |
| 26 | (Letter or Comment or Editorial or Case Reports).pt. | 4,023,224 |
| 27 | 25 not 26 | 1,507 |
| 28 | (animals not (animals and humans)).sh. | 4,890,387 |
| 29 | 27 not 28 | 1,480 |
| 30 | limit 29 to (danish or english or norwegian or swedish) | 1,400 |

**exp/** = term from the Medline controlled vocabulary, including terms found below this term in the MeSH hierarchy

**/** = term from the Medline controlled vocabulary, does not include terms found below this term in the MeSH hierarchy

**.ti,ab,kf**. = title, abstract and author keywords

**adjx** = within x words, regardless of order

***** = truncation of word for alternate endings

**Database:** EMBASE (OVID)

**Date:** 2021-12-03

**No of results:** 1400 ref

| **#** | **Search** | **Results** |
| --- | --- | --- |
| 1 | exp Anterior Cruciate Ligament/ | 12,658 |
| 2 | ACL.ab,kw,ti. | 23,963 |
| 3 | (anterior adj5 cruciate adj5 ligament*).ab,kw,ti. | 24,780 |
| 4 | 1 or 2 or 3 | 35,621 |
| 5 | (surgical* or surgery or surgeries or reconstruction* or reconstructive or reconstructed or repair).ab,kw,ti. | 3,192,584 |
| 6 | 4 and 5 | 21,399 |
| 7 | exp Anterior Cruciate Ligament Reconstruction/ | 12,609 |
| 8 | 6 or 7 | 22,923 |
| 9 | *hamstring tendon/ | 308 |
| 10 | *hamstring muscle/ | 764 |
| 11 | 9 or 10 | 1,066 |
| 12 | (Hamstring or semitendinosus).ab,kw,ti. | 12,065 |
| 13 | (graft or autograft or transplant* or tissue).ab,kw,ti. | 2,761,633 |
| 14 | 12 and 13 | 4,389 |
| 15 | 11 or 14 | 5,061 |
| 16 | exp treatment outcome/ | 1,879,669 |
| 17 | exp "Recovery of Function"/ | 52,815 |
| 18 | exp "Range of Motion, Articular"/ | 99,210 |
| 19 | ("range of motion" or recover* or outcome or function* or flexion or extension).ab,kw,ti. | 7,165,355 |
| 20 | 16 or 17 or 18 or 19 | 8,316,698 |
| 21 | (Muscle or flexor).ab,kw,ti. | 859,090 |
| 22 | strength.ab,kw,ti. | 377,207 |
| 23 | 21 and 22 | 65,993 |
| 24 | 20 or 23 | 8,340,819 |
| 25 | 8 and 15 and 24 | 2,069 |
| 26 | limit 25 to (books or conference abstract or "conference review" or editorial or erratum or letter or short survey or tombstone) | 324 |
| 27 | 25 not 26 | 1,745 |
| 28 | (animal not (animal and human)).sh. | 1,125,235 |
| 29 | 27 not 28 | 1,733 |
| 30 | limit 29 to (danish or english or norwegian or swedish) | 1,593 |

**exp/** = term from the Medline controlled vocabulary, including terms found below this term in the MeSH hierarchy

**/** = term from the Medline controlled vocabulary, does not include terms found below this term in the MeSH hierarchy

**.ti,ab,kf**. = title, abstract and author keywords

**adjx** = within x words, regardless of order

***** = truncation of word for alternate endings

**Database:** The Cochrane Library
**Date:** 2021-12-10
**No of results:** 501 ref

*Cochrane reviews: 2
Cochrane protocols: 0
Trials: 499
Editorials: 0
Special collections: 0
Clinical answers: 0*

| **ID** | **Search** | **Hits** |
| --- | --- | --- |
| #1 | MeSH descriptor: [Anterior Cruciate Ligament] explode all trees | 670 |
| #2 | (ACL):ti,ab,kw | 2,154 |
| #3 | ("anterior cruciate ligament"):ti,ab,kw | 2,846 |
| #4 | #1 OR #2 OR #3 | 3,292 |
| #5 | (surgical* or surgery or surgeries or reconstruction* or reconstructive or reconstructed or repair):ti,ab,kw | 273,197 |
| #6 | #4 AND #5 | 2,696 |
| #7 | MeSH descriptor: [Anterior Cruciate Ligament Reconstruction] explode all trees | 471 |
| #8 | #6 OR #7 | 2,697 |
| #9 | MeSH descriptor: [Hamstring Tendons] explode all trees and with  qualifier(s): [transplantation – TR] | 38 |
| #10 | MeSH descriptor: [Hamstring Muscles] explode all trees and with qualifier(s): [transplantation - TR] | 8 |
| #11 | #9 OR #10 | 44 |
| #12 | (Hamstring or semitendinosus):ti,ab,kw | 2,420 |
| #13 | (graft or autograft or transplant* or tissue):ti,ab,kw | 151,359 |
| #14 | #12 AND #13 | 788 |
| #15 | #11 OR #14 | 788 |
| #16 | MeSH descriptor: [Treatment Outcome] explode all trees | 148,566 |
| #17 | MeSH descriptor: [Recovery of Function] explode all trees | 5,583 |
| #18 | MeSH descriptor: [Range of Motion, Articular] explode all trees | 5,185 |
| #19 | ("range of motion" or recover* or outcome or function* or flexion or extension):ti,ab,kw | 756,616 |
| #20 | #16 OR #17 OR #18 OR #19 | 761,170 |
| #21 | (muscle OR flexor):ti,ab,kw | 79,751 |
| #22 | (Strength):ti,ab,kw | 39,858 |
| #23 | #21 AND #22 | 22,020 |
| #24 | #20 OR #23 | 766,520 |
| #25 | #8 AND #15 AND #24 | 501 |
|  | |  |

**Database:** AMED

**Date:** 2021-12-13

**No of results:** 102 ref

| **Search ID#** | **Search terms** | **Results** |
| --- | --- | --- |
| S23 | S6 AND S13 AND S20 - Limiters – Narrow by publication type: Academic journals,  Search modes - Find all my search terms | 102 |
| S22 | S6 AND S13 AND S20 - Limiters - Narrow by Language: - english  Search modes - Find all my search terms | 117 |
| S21 | S6 AND S13 AND S20 | 120 |
| S20 | S16 OR S19 | 91,123 |
| S19 | S17 AND S18 | 8,946 |
| S18 | TI strength OR AB strength OR KW strength | 13,788 |
| S17 | TI ( Muscle or flexor ) OR AB ( Muscle or flexor ) OR KW ( Muscle or flexor ) | 28,578 |
| S16 | S14 OR S15 | 87,341 |
| S15 | TI ( "range of motion" or recover* or outcome or function* or flexion or extension )  OR AB ( "range of motion" or recover* or outcome or function* or flexion or  extension ) OR KW ( "range of motion" or recover* or outcome or function* or  flexion or extension ) | 77,989 |
| S14 | (((ZU "treatment outcome")) or ((ZU "recovery of function"))) or  ((ZU "range of motion")) | 24,289 |
| S13 | S11 AND S12 | 238 |
| S12 | S9 OR S10 | 1,499 |
| S11 | TI ( graft or autograft or transplant* or tissue ) OR AB ( graft or autograft or  transplant* or tissue ) OR KW ( graft or autograft or transplant* or tissue ) | 10,645 |
| S10 | TI ( Hamstring or semitendinosus ) OR AB ( Hamstring or semitendinosus ) OR  KW ( Hamstring or semitendinosus ) | 1,499 |
| S9 | (ZU "hamstring muscles") | 139 |
| S8 | S6 OR S7 | 1,320 |
| S7 | (ZU "anterior cruciate ligament reconstruction") | 506 |
| S6 | S4 AND S5 | 1,320 |
| S5 | TI ( surgical* or surgery or surgeries or reconstruction* or reconstructive or  reconstructed or repair ) OR AB ( surgical* or surgery or surgeries or  reconstruction* or reconstructive or reconstructed or repair ) OR KW ( surgical*  or surgery or surgeries or reconstruction* or reconstructive or reconstructed or  repair ) | 16,201 |
| S4 | S1 OR S2 OR S3 | 2,341 |
| S3 | TI ( anterior N5 cruciate N5 ligament* ) OR AB ( anterior N5 cruciate N5 ligament* )  OR KW ( anterior N5 cruciate N5 ligament* ) | 2,280 |
| S2 | TI ACL OR AB ACL OR KW ACL | 1,105 |
| S1 | (ZU "anterior cruciate ligament") | 1,624 |
| ** TI betyder title, AB betyder abstract, ZU betyder indexerat ord, N5 betyder att det kan finnas max fem ord  mellan orden.* | | |

**Database:** PedRO

**Date:** 2021-12-13

**No of results:** 9

Anterior AND Cruciate AND Ligament AND reconstruction AND outcome = 9 studies
