# Supplementary material for: Comparison of knee flexor strength recovery between semitendinosus alone versus semitendinosus with gracilis autograft for ACL reconstruction: a systematic review and meta-analysis
Source: BMC Musculoskelet Disord. 2024 Feb 12;25:136. doi: 10.1186/s12891-024-07226-2 (PMC10863077; doi:10.1186/s12891-024-07226-2)
Supplement: Supplementary file 2 — Additional file 2. Supplemental file 2. [file 12891_2024_7226_MOESM2_ESM.docx]

**Supplemental file 2**

Second search in January 2023.

**Database:** MEDLINE (OVID)

**Date:** 2023-01-10

**No of results:** 1632 ref

| 1 | exp Anterior Cruciate Ligament/ | 12067 |
| --- | --- | --- |
| 2 | ACL.ab,kf,ti. | 20188 |
| 3 | (anterior adj5 cruciate adj5 ligament*).ab,kf,ti. | 23272 |
| 4 | 1 or 2 or 3 | 30392 |
| 5 | (surgical* or surgery or surgeries or reconstruct* or repair*).ab,kf,ti. | 2705772 |
| 6 | 4 and 5 | 19043 |
| 7 | exp Anterior Cruciate Ligament Reconstruction/ | 7084 |
| 8 | 6 or 7 | 19493 |
| 9 | Hamstring Tendons/tr | 432 |
| 10 | Hamstring Muscles/tr | 66 |
| 11 | 9 or 10 | 493 |
| 12 | Hamstring Tendons/ or Hamstring Muscles/ | 1996 |
| 13 | (Hamstring* or semitendinos*).ab,kf,ti. | 13171 |
| 14 | 12 or 13 | 13462 |
| 15 | (graft* or autograft* or transplant* or tissue).ab,kf,ti. | 2318059 |
| 16 | 14 and 15 | 4587 |
| 17 | 11 or 16 | 4614 |
| 18 | exp "Recovery of Function"/ | 58853 |
| 19 | exp "Range of Motion, Articular"/ | 59227 |
| 20 | ("range of motion" or recover* or function* or flexion or extension).ab,kf,ti. | 5130353 |
| 21 | 18 or 19 or 20 | 5160820 |
| 22 | (Muscle or flexor).ab,kf,ti. | 739289 |
| 23 | strength.ab,kf,ti. | 361494 |
| 24 | 22 and 23 | 53844 |
| 25 | 21 or 24 | 5183869 |
| 26 | 8 and 17 and 25 | 1570 |
| 27 | exp Reinjuries/ | 129 |
| 28 | (re-injur* or reinjur* or rerupt*).ab,kf,ti. | 2833 |
| 29 | (second* or Subsequen*).ab,kf,ti. | 2837104 |
| 30 | 27 or 28 or 29 | 2839344 |
| 31 | 8 and 17 and 30 | 575 |
| 32 | 26 or 31 | 1839 |
| 33 | (animals not (animals and humans)).sh. | 5046442 |
| 34 | 32 not 33 | 1804 |
| 35 | (Letter or Comment or Editorial or Case Reports).pt. | 4207028 |
| 36 | 34 not 35 | 1721 |
| 37 | limit 36 to (danish or english or norwegian or swedish) | 1632 |

**exp/** = term from the Medline controlled vocabulary, including terms found below this term in the hierarchy

**/** = term from the Medline controlled vocabulary, does not include terms found below this term in the hierarchy

**.ti,ab,kf**. = title, abstract and author keywords

**adjx** = within x words, regardless of order

***** = truncation of word for alternate endings

**Database:** EMBASE (OVID)

**Date:** 2023-01-10

**No of results:** 2962 ref

| 1 | exp anterior cruciate ligament/ | 13327 |
| --- | --- | --- |
| 2 | ACL.ab,kf,ti. | 26813 |
| 3 | (anterior adj5 cruciate adj5 ligament*).ab,kf,ti. | 27747 |
| 4 | 1 or 2 or 3 | 38911 |
| 5 | (surgical* or surgery or surgeries or reconstruct* or repair*).ab,kf,ti. | 3507927 |
| 6 | 4 and 5 | 23712 |
| 7 | exp Anterior Cruciate Ligament Reconstruction/ | 14577 |
| 8 | 6 or 7 | 25495 |
| 9 | exp hamstring tendon/ | 1256 |
| 10 | exp hamstring muscle/ | 8925 |
| 11 | (Hamstring* or semitendinos*).ab,kf,ti. | 15999 |
| 12 | 9 or 10 or 11 | 19422 |
| 13 | (graft* or autograft* or transplant* or tissue).ab,kf,ti. | 3073895 |
| 14 | 12 and 13 | 6202 |
| 15 | exp convalescence/ | 56262 |
| 16 | exp "joint characteristics and functions"/ | 104583 |
| 17 | ("range of motion" or recover* or function* or flexion or extension).ab,kf,ti. | 6372937 |
| 18 | 15 or 16 or 17 | 6433716 |
| 19 | (Muscle or flexor).ab,kf,ti. | 920300 |
| 20 | strength.ab,kf,ti. | 414438 |
| 21 | 19 and 20 | 74970 |
| 22 | 18 or 21 | 6464590 |
| 23 | 8 and 14 and 22 | 2119 |
| 24 | exp Reinjuries/ | 2518395 |
| 25 | (re-injur* or reinjur* or rerupt*).ab,kf,ti. | 3416 |
| 26 | (second* or subsequen*).ab,kf,ti. | 3787381 |
| 27 | 24 or 25 or 26 | 5965677 |
| 28 | 8 and 14 and 27 | 2542 |
| 29 | 23 or 28 | 3228 |
| 30 | animal/ not (animal/ and human/) | 1172192 |
| 31 | 29 not 30 | 3192 |
| 32 | (comment or editorial or letter).pt. | 2001518 |
| 33 | 31 not 32 | 3174 |
| 34 | limit 33 to (danish or english or norwegian or swedish) | 2962 |

**exp/** = term from the Emtree controlled vocabulary, including terms found below this term in the hierarchy

**/** = term from the Medline controlled vocabulary, does not include terms found below this term in thehierarchy

**.ti,ab,kf**. = title, abstract and author keywords

**adjx** = within x words, regardless of order

***** = truncation of word for alternate endings

**Database:** The Cochrane Library
**Date:** 2023-01-10
**No of results:** 365 ref

*Cochrane reviews: 1
Cochrane protocols: 0
Trials: 364
Editorials: 0
Special collections: 0*

| #1 | MeSH descriptor: [Anterior Cruciate Ligament] explode all trees | 688 |
| --- | --- | --- |
| #2 | (ACL):ti,ab,kw | 2411 |
| #3 | (anterior near/5 cruciate near/5 ligament*):ti,ab,kw | 3167 |
| #4 | #1 OR #2 OR #3 | 3636 |
| #5 | (surgical* or surgery or surgeries or reconstruct* or repair*):ti,ab,kw | 299578 |
| #6 | #4 AND #5 | 3001 |
| #7 | MeSH descriptor: [Anterior Cruciate Ligament Reconstruction] explode all trees | 522 |
| #8 | #6 OR #7 | 3002 |
| #9 | MeSH descriptor: [Hamstring Tendons] explode all trees | 52 |
| #10 | MeSH descriptor: [Hamstring Muscles] explode all trees | 181 |
| #11 | (Hamstring* or semitendinos*):ti,ab,kw | 3214 |
| #12 | #9 OR #10 OR #11 | 3214 |
| #13 | (graft* or autograft* or transplant* or tissue):ti,ab,kw | 168198 |
| #14 | #12 AND #13 | 984 |
| #15 | MeSH descriptor: [Recovery of Function] explode all trees | 5740 |
| #16 | MeSH descriptor: [Range of Motion, Articular] explode all trees | 5387 |
| #17 | ("range of motion" or recover* or function* or flexion or extension):ti,ab,kw | 385685 |
| #18 | #15 OR #16 OR #17 | 385695 |
| #19 | (Muscle or flexor):ti,ab,kw | 88026 |
| #20 | (strength):ti,ab,kw | 44642 |
| #21 | #19 AND #20 | 24872 |
| #22 | #18 OR #21 | 394378 |
| #23 | #8 AND #14 AND #22 | 464 |
| #24 | MeSH descriptor: [Reinjuries] explode all trees | 8 |
| #25 | (re-injur* or reinjur* or rerupt*):ti,ab,kw | 478 |
| #26 | (second* or Subsequen*):ti,ab,kw | 481203 |
| #27 | #24 OR #25 OR #26 | 481465 |
| #28 | #8 AND #14 AND #27 | 233 |
| #29 | #23 OR #28 | 551 |
| #30 | (clinicaltrials OR trialsearch):so | 443182 |
| #31 | (conference proceeding):pt | 215178 |
| #32 | #30 OR #31 | 658360 |
| #33 | #29 NOT #32 | 365 |

**MeSH descriptor: [] explode all trees:** term from the MeSH controlled vocabulary, including terms found below this term in the hierarchy

**.ti,ab,kw**. = title, abstract and author keywords

**Near/** = within x words, regardless of order

***** = truncation of word for alternate endings

**Database:** AMED

**Date:** 2023-01-10

**No of results:** 100 ref

| S28 | S22 OR S26  Narrow by Language: - english | 100 |
| --- | --- | --- |
| S27 | S22 OR S26 | 117 |
| S26 | S8 AND S13 AND S25 | 27 |
| S25 | S23 OR S24 | 21,189 |
| S24 | TI ( second* or subsequen* ) OR AB ( second* or subsequen* ) OR KW ( second* or subsequen* ) | 20,925 |
| S23 | TI ( re-injur* or reinjur* or rerupt* ) OR AB ( re-injur* or reinjur* or rerupt* ) OR KW ( re-injur* or reinjur* or rerupt* ) | 330 |
| S22 | S8 AND S13 AND S21 | 111 |
| S21 | S17 OR S20 | 64,632 |
| S20 | S18 AND S19 | 9,306 |
|  |  |  |
|  |  |  |
| S19 | TI strength OR AB strength OR KW strength | 14,379 |
|  |  |  |
|  |  |  |
| S18 | TI ( Muscle or flexor ) OR AB ( Muscle or flexor ) OR KW ( Muscle or flexor ) | 29,735 |
| S17 | S14 OR S15 OR S16 | 60,117 |
| S16 | TI ( "range of motion" or recover* or function* or flexion or extension ) OR AB ( "range of motion" or recover* or function* or flexion or extension ) OR KW ( "range of motion" or recover* or function* or flexion or extension ) | 59,748 |
| S15 | (ZU "range of motion") | 5,246 |
|  |  |  |
|  |  |  |
| S14 | (ZU "recovery of function") | 1,205 |
| S13 | S11 AND S12 | 263 |
| S12 | S9 OR S10 | 1,597 |
| S11 | TI ( graft* or autograft* or transplant* or tissue ) OR AB ( graft* or autograft* or transplant* or tissue ) OR KW ( graft* or autograft* or transplant* or tissue ) | 11,538 |
| S10 | TI ( Hamstring* or semitendinos* ) OR AB ( Hamstring* or semitendinos* ) OR KW ( Hamstring* or semitendinos* ) | 1,597 |
| S9 | (ZU "hamstring muscles") | 177 |
| S8 | S6 OR S7 | 1,421 |
|  |  |  |
|  |  |  |
| S7 | (ZU "anterior cruciate ligament reconstruction") | 593 |
| S6 | S4 AND S5 | 1,421 |
| S5 | TI ( surgical* or surgery or surgeries or reconstruct* or repair* ) OR AB ( surgical* or surgery or surgeries or reconstruct* or repair* ) OR KW ( surgical* or surgery or surgeries or reconstruct* or repair* ) | 17,288 |
| S4 | S1 OR S2 OR S3 | 2,476 |
| S3 | TI ( anterior N5 cruciate N5 ligament* ) OR AB ( anterior N5 cruciate N5 ligament* ) OR KW ( anterior N5 cruciate N5 ligament* ) | 2,413 |
| S2 | TI ACL OR AB ACL OR KW ACL | 1,174 |
| S1 | (ZU "anterior cruciate ligament") | 1,672 |

**ZU:** term from the Amed controlled vocabulary, including terms found below this term in the hierarchy

**.ti,ab,kw**. = title, abstract and author keywords

**Nx** = within x words, regardless of order

***** = truncation of word for alternate endings

**Database:** PedRO

**Date:** 2023-01-10

**No of results:** 14

Pedro 1: Anterior AND Cruciate AND Ligament AND reconstruct* AND reinjur* = 3ref

Pedro 2: Anterior AND Cruciate AND Ligament AND reconstruct* AND subsequent injur* = 2 ref

Pedro 3: Anterior AND Cruciate AND Ligament AND reconstruction AND outcome = 9ref

***** = truncation of word for alternate endings
